# Experimental Evidence of Caffeic Acid’s Neuroprotective Activity in Alzheimer’s Disease: In Vitro, In Vivo, and Delivery-Based Insights

**DOI:** 10.3390/medicina61081428

**Published:** 2025-08-08

**Authors:** Adam Kowalczyk, Carlo Ignazio Giovani Tuberoso, Igor Jerković

**Affiliations:** 1 Department of Pharmacognosy and Herbal Medicines, Faculty of Pharmacy, Wroclaw Medical University, 50-556 Wrocław, Poland; 2 Unit of Pharmaceutical, Department of Life and Environmental Sciences, Pharmacological and Nutraceutical Sciences, University of Cagliari, 09042 Monserrato, Italy; tuberoso@unica.it; 3 Mediterranean Institute for Life Sciences, University of Split, 21000 Split, Croatia

**Keywords:** caffeic acid, Alzheimer’s disease, in vitro, in vivo, neuroprotection, drug delivery

## Abstract

*Background and Objectives*: Alzheimer’s disease (AD) is a complex neurodegenerative disorder marked by cholinergic deficits, oxidative stress, amyloid-β (Aβ) aggregation, and tau hyperphosphorylation. Caffeic acid (CA), a naturally occurring hydroxycinnamic acid, has emerged as a promising neuroprotective candidate due to its antioxidant, anti-inflammatory, and enzyme-inhibitory properties. This review systematically evaluates recent in vitro and in vivo evidence regarding the therapeutic potential of CA in AD models and examines the impact of delivery systems and derivatives on its efficacy and bioavailability. *Materials and Methods*: A systematic literature search was conducted in the PubMed, Scopus, and Web of Science databases, adhering to the PRISMA 2020 guidelines. Studies published between January 2021 and April 2025 were included in this review. Eligible studies investigated the effects of CA or CA-enriched extracts on AD-relevant mechanisms using in vitro, in vivo, and in silico models. After screening 101 articles, 44 met the inclusion criteria and were included in the final qualitative synthesis of the study. *Results:* In vitro studies have confirmed that CA modulates cholinergic activity by inhibiting AChE and BChE and exerting antioxidant and anti-amyloidogenic effects. In vivo studies using pharmacological, genetic, and metabolic AD models have demonstrated improvements in cognitive function, reduction in oxidative stress, inflammation, and Aβ and tau pathologies following CA administration. Advanced delivery platforms, such as solid lipid nanoparticles, transferrin-functionalized liposomes, and carbon dot systems, have significantly enhanced CA’s brain bioavailability and therapeutic efficacy. CA derivatives, including caffeic acid phenethyl ester and nitro-substituted analogs, exhibit improved pharmacokinetic and neuroprotective profiles. *Conclusions:* This review provides evidence supporting the use of CA as a promising multitarget agent against AD pathology. Its therapeutic potential is further enhanced by nanotechnology-based delivery systems and chemical modifications that overcome the limitations of bioavailability. Continued preclinical evaluation and translational studies are warranted to support its clinical development as an AD intervention.

## 1. Introduction

Alzheimer’s disease (AD) is the most prevalent neurodegenerative disorder globally, constituting a significant public health challenge, particularly among the elderly population [1]. Clinically, AD is characterized by a progressive deterioration in memory, executive function, language, and orientation. Pathologically, the disease is marked by the extracellular deposition of β-amyloid (Aβ) plaques, intracellular accumulation of hyperphosphorylated tau protein into neurofibrillary tangles, chronic neuroinflammation, oxidative stress, synaptic dysfunction, and ultimately, neuronal loss [2,3]. These pathological features collectively disrupt neural networks and impair cognitive function. Current pharmacological interventions for AD encompass cholinesterase inhibitors such as donepezil and rivastigmine, which mitigate cholinergic deficits, and NMDA (N-methyl-D-aspartate) receptor antagonists such as memantine, which modulate excitotoxic glutamatergic signaling. In the past several years, therapeutic strategies targeting Aβ have garnered increasing interest. Monoclonal antibodies, such as aducanumab and lecanemab, have been developed to selectively bind to and facilitate the clearance of Aβ aggregates. Although these therapies have demonstrated some efficacy in decelerating disease progression, they remain contentious due to limited clinical benefits and concerns regarding safety profiles, including the risk of amyloid-related imaging abnormalities (ARIA). These limitations underscore the necessity for novel neuroprotective strategies that address the multifaceted pathological pathways of AD [4,5].

**Figure 1:**
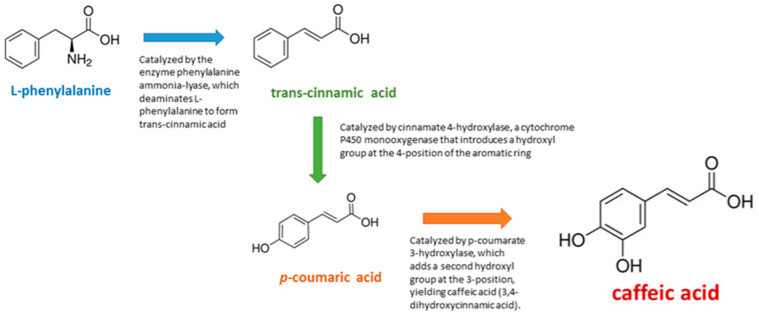
Key steps of CA biosynthesis.

There has been an increasing focus on identifying disease-modifying strategies that concurrently address multiple pathological mechanisms. Natural products, particularly polyphenolic compounds, have emerged as promising candidates for the treatment of neurodegeneration due to their capacity to modulate diverse cellular pathways implicated in the disease [6]. Among these compounds, caffeic acid (CA) has gained recognition for its broad-spectrum bioactivity, low toxicity, and presence in commonly consumed plant-based foods [7]. CA is a hydroxycinnamic acid derived from the phenylpropanoid pathway in plants and is primarily synthesized through the hydroxylation of *p*-coumaric acid [8]. Figure 1 illustrates the key steps of its biosynthesis. It is ubiquitously distributed in the human diet, with rich sources including coffee, berries, grapes, apples, pears, and various medicinal herbs [7].

At the molecular level, CA demonstrates several neuroprotective effects pertinent to the treatment of AD. These effects include significant antioxidant activity, which is attributed to its catechol structure, enabling it to effectively scavenge reactive oxygen species (ROS) and chelate redox-active metals such as iron and copper [6,8]. Additionally, CA exhibits anti-inflammatory properties by modulating key signaling pathways, including NF-κB, MAPK, and Nrf2 (nuclear factor erythroid 2–related factor 2), thereby suppressing the expression of proinflammatory cytokines (e.g., TNF-α, IL-1β) and enzymes (e.g., COX-2, iNOS). Furthermore, CA has been shown to inhibit Aβ aggregation, reduce tau phosphorylation, and enhance synaptic plasticity, all of which are critical targets for mitigating the progression of AD [6,9,10].

**Figure 2:**
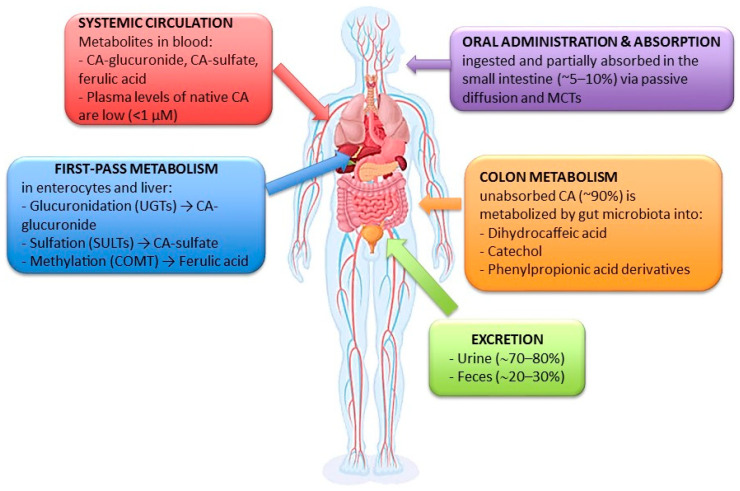
Critical stages in the pharmacokinetics and metabolic disposition of CA following oral administration in humans.

Despite its promising therapeutic potential, CA’s clinical application is constrained by its pharmacokinetic characteristics. CA is absorbed in the small intestine through passive diffusion and monocarboxylate transporters (MCTs), albeit at a limited rate of approximately 5–10%. The majority of ingested CA, around 90%, proceeds to the colon, where it undergoes microbial transformation into various metabolites, including dihydrocaffeic acid, catechol, and phenylpropionic acid derivatives. During first-pass metabolism in enterocytes and the liver, CA is subjected to enzymatic conjugation processes: glucuronidation by UDP-glucuronosyltransferases (UGTs) results in CA-glucuronide, sulfation by sulfotransferases (SULTs) produces CA-sulfate, and methylation by catechol-O-methyltransferase (COMT) leads to ferulic acid. In systemic circulation, CA predominantly exists as conjugated metabolites, such as CA-glucuronide and CA-sulfate, with native CA present at very low plasma concentrations (<1 µM). Excretion primarily occurs via urine, accounting for approximately 70–80%, and to a lesser extent, via feces, comprising approximately 20–30% [8,11]. Figure 2 illustrates the principal pharmacokinetic processes and metabolic transformations of CA following oral administration in humans. Its hydrophilic nature and moderate molecular weight further restrict its permeability across the blood–brain barrier (BBB), a significant challenge in central nervous system (CNS) drug development [12,13]. Consequently, there is increasing interest in enhancing CA’s bioavailability and brain-targeting capability through innovative drug delivery systems [14,15].

To date, no comprehensive review has synthesized recent findings on the neuroprotective potential of CA against AD by integrating evidence from in vitro and in vivo studies with innovations in delivery systems. This review addresses this gap by systematically summarizing the mechanistic insights into CA’s effects on cholinergic neurotransmission, oxidative stress, amyloid and tau pathologies, and neuroinflammation. Notably, it incorporates advanced molecular modeling data, such as docking and molecular dynamics simulations, highlighting CA’s multitarget pharmacological profile. Furthermore, this study provides an updated perspective on CA’s limited bioavailability and recent solutions based on nanoformulations and targeted delivery approaches. Covering research published between 2021 and 2025, this work delivers a timely and comprehensive overview of CA’s therapeutic potential in AD, offering a robust foundation for future research. The collected findings were categorized into three principal domains: (1) in vitro studies that elucidate CA’s molecular targets and signaling pathways; (2) in vivo studies that demonstrate behavioral, biochemical, and histopathological benefits in pharmacological and transgenic models of AD; (3) formulation strategies aimed at improving bioavailability and central nervous system penetration. The critical analysis of this integrated evidence base underscores the promise of CA as a multifunctional neuroprotective compound and supports its further investigation and development as a therapeutic candidate for AD.

## 2. Materials and Methods

**Figure 3:**
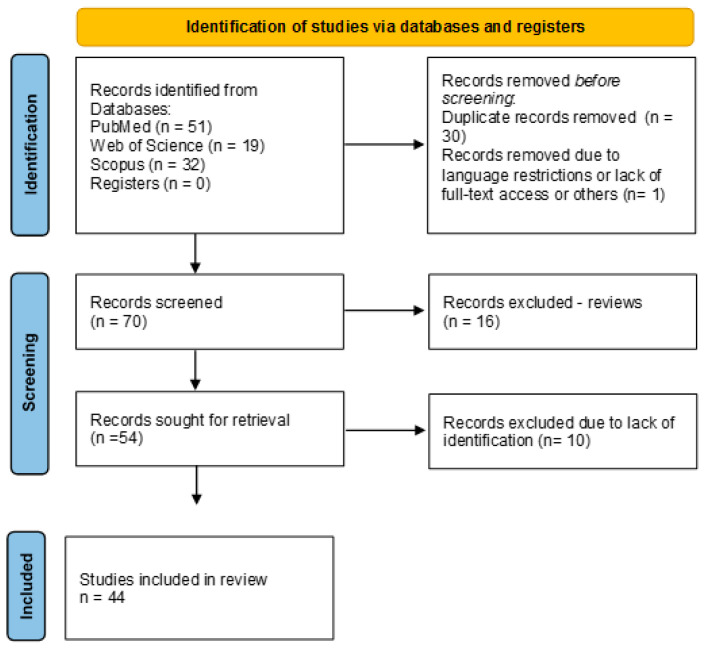
PRISMA flowchart of the included studies.

This systematic review was conducted in accordance with the Preferred Reporting Items for Systematic Reviews and Meta-Analyses (PRISMA 2020) guidelines to ensure methodological transparency and reproducibility. The completed PRISMA checklist is provided in the Supplementary Materials (Table S1), and the PRISMA flow diagram is presented in Figure 3. This review was not registered in a public database. A comprehensive literature search was performed using the PubMed, Scopus, and Web of Science databases to identify studies investigating the neuroprotective effects of CA in the context of AD. Searches were completed in April 2025 and included articles published between January 2021 and April 2025. The following combinations of keywords: “caffeic acid” AND “Alzheimer’s disease,” “hydroxycinnamic acid” AND “neuroprotection,” “caffeic acid” AND “oxidative stress,” “caffeic acid” AND “cholinesterase inhibition,” and “caffeic acid” AND “amyloid beta” OR “tau protein.” Only original studies published in English with full-text availability were included in this review. Studies were eligible if they reported on in vitro, in vivo, or clinical experimental models related to AD and demonstrated the mechanistic or therapeutic activity of CA or CA-enriched extracts. Review articles, conference abstracts, editorials, and studies unrelated to AD pathology were excluded from the study. Duplicates and retracted articles were removed before screening. The authors screened the titles, abstracts, and full texts. Disagreements were resolved by consensus or consultation with a third reviewer. Of the 101 initially identified articles, 30 duplicates and 1 non-English record were excluded. After screening, 70 unique articles remained, and 16 review papers were excluded. After the full-text assessment of 54 studies, an additional 10 were excluded due to insufficient mechanistic relevance or methodological limitations, resulting in the final inclusion of 44 eligible studies. The flow of study selection is summarized in the PRISMA diagram (Figure 3), detailing the number of records identified, screened, and ultimately included in the qualitative synthesis of the studies.

## 3. Results

To ensure clarity and prevent redundancy, each publication was categorized into one of three distinct groups: in vitro studies, which primarily focus on cellular assays, enzymatic tests, and molecular simulations; in vivo studies, which involve the use of animals or model organisms to evaluate the cognitive and biochemical effects of CA; and delivery systems and derivatives, which aim to enhance CA’s bioavailability and permeability across the BBB through formulation strategies. This categorization reflects the primary objective of each study and ensures consistency in data interpretation.

### 3.1. In Vitro Studies

Cholinergic deficits constitute a fundamental component of AD pathology, primarily attributable to the progressive degeneration of cholinergic neurons and the subsequent reduction in acetylcholine (ACh) levels within the cortex and hippocampus. The therapeutic rationale for cholinesterase inhibition is based on the restoration of cholinergic neurotransmission by preventing the degradation of ACh. Although AChE is the predominant enzyme under physiological conditions, butyrylcholinesterase (BChE) assumes a compensatory function as AD advances, making dual inhibition of both enzymes a more comprehensive and clinically relevant strategy. CA and CA-rich plant extracts have been demonstrated to modulate cholinergic pathways through direct enzymatic inhibition or indirect signaling regulation [16,17,18].

CA has emerged as a promising modulator of cholinergic neurotransmission, with multiple studies highlighting its selective and dual inhibitory effects on key enzymes, such as AChE and BChE. Extracts enriched in CA, including those derived from *Ononis natrix* and *Laurus nobilis*, have demonstrated potent BChE inhibition, with IC_50_ values comparable to those of rivastigmine, particularly in advanced AD, where BChE activity surpasses that of AChE [19,20]. These findings suggest the therapeutic viability of CA-rich phytochemicals and the potential role of extraction and thermal processing in enhancing their bioactivity. Further reinforcing CA’s cholinergic effects, polyphenolic preparations such as propolis extracts, containing high CA concentrations (21.36 mg/g), exhibited significant AChE inhibition (IC_50_ = 3.4 µg/mL), consistent with the effects observed for clinically established inhibitors [21]. The dual inhibition profile was echoed in studies using *Beta vulgaris* extracts, where moderate AChE and BChE inhibition was supported by molecular docking and dynamic simulations. These simulations revealed the stable interactions of CA with enzymatically active sites, underscoring its polypharmacological potential in multitarget AD therapy [22]. In addition to direct enzymatic inhibition, CA may indirectly influence cholinergic signaling through upstream regulatory pathways. For instance, although present at lower concentrations, CA was identified as a contributor to GSK-3β inhibition in *Salvia fruticosa* infusion, an effect with implications for both cholinergic function and tau pathology. Docking analyses suggest that CA participates in modulating kinase-driven mechanisms of neurodegeneration [23].

#### 3.1.1. Antioxidant and Redox Modulation

Oxidative stress is a pivotal factor in the pathogenesis of AD, facilitating neuronal damage through the overproduction of ROS, mitochondrial dysfunction, and neuroinflammation. CA, due to its catechol moiety, has exhibited significant antioxidant properties in various in vitro models, both by directly scavenging radicals and by indirectly modulating redox-sensitive signaling pathways [24].

The antioxidant properties of CA and CA-enriched extracts have been substantiated through a range of chemical assays and cell-based models, with increasing evidence associating their redox activity with neuroprotection in AD. Numerous studies have demonstrated that CA-containing botanical extracts, such as those from *Sorghum bicolor*, *Blumea laciniata*, and *Camellia sinensis*, exhibit significant radical scavenging capacity, as evaluated by DPPH, ABTS, CUPRAC, and FRAP assays. Fermentation and other post-harvest processing techniques appear to enhance the redox potential and cholinesterase-inhibitory activity of these preparations, suggesting the functional optimization of CA bioactivity through processing [25,26,27]. Beyond chemical assays, CA’s impact extends to cellular models pertinent to AD. CA-rich extracts have been shown to mitigate β-amyloid-induced oxidative stress, maintain mitochondrial function, and inhibit apoptosis in human neuroblastoma and murine hippocampal neuron-like cell lines. These protective effects are mediated via key antioxidant and pro-survival signaling pathways, including PI3K/Akt and MAPK. Systems pharmacology and molecular docking analyses have further revealed that CA interacts with molecular targets such as COX-2 (PTGS2), MMP9, and MAPK14, reinforcing its role in modulating redox-sensitive and inflammatory responses [28,29]. These outcomes converge on a dual mechanism through which CA exerts neuroprotective effects as a direct ROS scavenger and regulator of redox-sensitive transcriptional networks and enzymes. This multi-level redox modulation underscores CA’s therapeutic potential in mitigating oxidative stress-driven neurodegeneration, a hallmark of AD.

#### 3.1.2. Anti-Amyloid and Anti-Tau Effects

The accumulation of Aβ plaques and hyperphosphorylated tau tangles are recognized as pathological hallmarks of AD, contributing to synaptic failure, mitochondrial dysfunction, and neuronal loss. Therapeutic interventions targeting both Aβ aggregation and tau hyperphosphorylation are essential for halting or reversing neurodegeneration. CA, due to its polyphenolic structure, has demonstrated promising activity in modulating these pathological events through both direct molecular interactions [30,31].

Recent investigations have broadened our comprehension of CA’s multifaceted neuroprotective properties by exploring its impact on tau pathology, mitochondrial integrity, and target selectivity in both in silico and cellular environments. Extracts rich in CA from *Sorghum bicolor* have demonstrated the ability to mitigate tau hyperphosphorylation (notably at pS199 and pT231) and restore mitochondrial function in Aβ_42_-stressed neuroblastoma cells, indicating a dual mechanism involving anti-aggregation and bioenergetic stabilization [32]. Complementary computational analyses have identified CA as a dual-target ligand for AChE and NMDARs, two pivotal proteins in cholinergic and excitotoxic signaling, underscoring its potential for multitarget modulation in AD, despite not consistently being the most potent binder [33]. These results underscore the necessity for context-specific validation. In a ligand fishing study utilizing AChE-functionalized magnetic nanoparticles, CA did not exhibit direct binding, highlighting the limitations of simplified models and reinforcing the importance of analyzing whole-extract effects in biologically relevant environments [34]. Other studies have supported this integrative perspective. In *Fabaceae*-derived leaf extracts, CA was the predominant phenolic constituent and was associated with significant AChE inhibition and antioxidant capacity, even though its isolated contribution was not quantified, further emphasizing the complexity of extract-based interventions [35]. Mechanistic synergies between CA and other compounds also emerge in these polyherbal formulations. In a neuroprotective preparation containing huperzine A, CA enhanced efficacy against glutamate- and Aβ-induced toxicity without increasing cholinergic side effects, acting via ERK1/2 pathway activation rather than direct cholinesterase inhibition [36]. Further molecular insights have confirmed CA’s binding compatibility with AChE, ApoE4, and transferrin, as demonstrated in advanced docking and molecular dynamics studies [37,38,39]. These computational data suggest that CA plays a role in modulating iron metabolism, genetic risk-related pathways, and enzyme inhibition, positioning it as a versatile neuroprotective agent in the AD therapeutic landscape.

### 3.2. In Vivo Studies

In vivo investigations have yielded critical evidence regarding the therapeutic potential of CA in animal models of AD. These models facilitate the evaluation of behavioral, biochemical, and histopathological outcomes following CA administration. For enhanced clarity, the studies are categorized according to experimental model systems: pharmacologically induced AD models, transgenic or genetic models, and dietary or metabolic models [40].

#### 3.2.1. Pharmacological Models of AD

Pharmacological models of AD, such as those induced by scopolamine, trimethyltin (TMT), sodium azide, or D-galactose with aluminum chloride, are extensively utilized to replicate key neurodegenerative processes, including cholinergic dysfunction, oxidative stress, and β-amyloid accumulation. These models offer a controlled environment for assessing neuroprotective compounds [41]. Numerous studies have employed these models to investigate the efficacy of CA or CA-enriched formulations.

Latest in vivo studies have substantiated the ability of CA and CA-enriched botanical preparations to ameliorate memory deficits and neuropathological changes in various pharmacological models of Alzheimer’s disease (AD). In the context of trimethyltin (TMT)-induced hippocampal neurotoxicity, CA and ethanol extracts of *Ixeris dentata* have been shown to enhance spatial learning and memory, potentially through the upregulation of ChAT and CREB in hippocampal neurons, thereby indicating improved cholinergic signaling and synaptic plasticity [42]. Similarly, in scopolamine-induced amnesia, polyherbal formulations such as Divya-Medha-Vati, which includes CA among other phytochemicals, have been observed to reverse behavioral impairments, mitigate oxidative stress, and elevate the expression of neurotrophic genes (BDNF, NGF-1, SYN-1), suggesting that CA may facilitate the activation of neurogenic pathways in addition to its antioxidant properties [43]. Other research has highlighted the significance of CA in broader anti-inflammatory and amyloid-modulating effects. In a sodium azide-induced AD model, *Mazus pumilus* methanolic extract has been found to improve cognitive outcomes, reduce AChE activity, and diminish amyloid plaque deposition, further corroborating the multifaceted efficacy of CA-containing phytochemicals [44]. These effects are mirrored in the use of *Desmodium elegans* extract, which, through integrated in vitro, in silico, and in vivo approaches, has demonstrated dual cholinesterase inhibition, antioxidant capacity, and anxiolytic-like effects, closely aligning with the activity profile of CA-rich polyphenolic formulations [45]. Metabolomic profiling enhances mechanistic understanding. In a D-galactose and aluminum chloride-induced AD model, the ethyl acetate fraction of *Physalis alkekengi* fruit extract—rich in CA, nobiletin, and physalin B—has shown superior efficacy in reducing Aβ and tau burdens while modulating neuroinflammatory markers and suppressing p38 MAPK signaling. These insights not only associate CA with anti-amyloid and anti-inflammatory activity but also suggest its potential to modulate intracellular kinase cascades linked to neurodegeneration [46].

#### 3.2.2. Transgenic and Genetic Models

Transgenic and genetic models of AD provide essential platforms for evaluating the multifaceted neuroprotective effects of CA in vivo. These models replicate key pathological features of AD, including Aβ aggregation, tau hyperphosphorylation, and cognitive deficits, thereby allowing for a detailed investigation of CA’s mechanistic actions of CA under disease-relevant conditions.

Complementary in vivo studies utilizing both mammalian and non-mammalian models have significantly enhanced our comprehension of CA’s multifaceted neuroprotective properties. In ApoE−/− mice, which serve as a dual model for atherosclerosis and AD, the administration of CA markedly improved spatial memory and reduced hippocampal Aβ burden, while simultaneously decreasing systemic and cerebral inflammation. These effects were mechanistically associated with increased expression of lipid transporters (ABCA1/ABCG1) and a reduction in proinflammatory cytokines (TNF-α, IL-6, and MCP-1), thereby supporting a combined anti-amyloid and vascular-protective mechanism of action [47]. The significance of CA in complex botanical preparations has been further corroborated through systems pharmacology analyses. In a scopolamine-induced AD model, CA-enriched *Acanthopanax senticosus* extract restored cholinergic function and inhibited hippocampal apoptosis, demonstrating multitarget efficacy as confirmed through interaction network mapping [48]. In lower organisms, the neuroprotective efficacy of CA has been validated in both *Drosophila* and *Caenorhabditis elegans*. In transgenic flies expressing human APP and BACE1, dietary CA-rich *Solanum* extracts improved behavioral and survival outcomes, reduced Aβ and MAO activities, and mitigated oxidative stress [49]. Similarly, in *Caenorhabditis elegans*, CA delayed Aβ-induced paralysis and enhanced stress resilience via the DAF-16/FOXO pathway, leading to increased expression of antioxidant, proteostatic, and heat shock response genes [50]. These conclusions underscore the conserved nature of CA’s mechanisms across species and its potential to modulate core cellular pathways involved in protein aggregation, oxidative injury, and neurodegeneration.

#### 3.2.3. Dietary and Metabolic Models

A substantial body of research has increasingly concentrated on the translational potential of CA through various delivery methods and dietary sources, as well as its interaction with multiple molecular targets in AD models. For instance, Khan et al. [8] demonstrated that oral administration of CA enhances BDNF expression and PI3K/Akt signaling in Aβ_1–42_-treated mice, leading to cognitive improvement and the suppression of neuroinflammation and oxidative stress. These in vivo effects are further corroborated by in vitro and in silico studies, where CA exhibited favorable binding to AD-relevant targets such as AChE and TACE (TNF-α converting enzyme), implicating it in both cholinergic and anti-inflammatory regulation [51,52]. The dietary relevance of this compound has also been established. Grzelczyk et al. [53] found that digested CA-rich fractions from coffee retained potent BChE inhibitory activity, suggesting the bioactivity of CA following gastrointestinal processing. Similarly, encapsulated phenolic-rich peanut meal extract containing CA demonstrated improved antioxidant status and neurotransmitter balance in AlCl_3_-induced AD rats, with alginate-based encapsulation optimizing delivery [52]. Metabolic comorbidities common in AD have been addressed in models where CA, although less potent than stingless bee honey, mitigated neuroinflammatory markers and metabolic dysfunction in a high-fructose-induced rat model, reinforcing its systemic protective effects [54]. These observations position CA as a promising multitarget agent whose efficacy can be modulated by formulation strategies, metabolic status, and dietary context.

### 3.3. Delivery Systems

Due to the limited bioavailability and restricted permeability of CA across the BBB, numerous studies have investigated novel delivery systems and chemical derivatives to enhance its therapeutic efficacy for AD.

### 3.4. Nanoformulations for Bioavailability Enhancement

Recent advancements in nanoformulation strategies have markedly enhanced the bioavailability and therapeutic efficacy of CA in AD models. For example, magnesium oxide nanocomposites, loaded with chitosan and polylactic acid and synthesized using *Coffea arabica* leaf extract, have demonstrated significant neuroprotective effects in a streptozotocin-induced rat model of diabetic neuropathy. These effects include improved spatial memory, reduced AChE activity, and restored redox and mitochondrial functions. Docking studies have confirmed CA’s strong binding affinity to Aβ fibrils, supporting its anti-amyloidogenic potential [55]. Similarly, chitosan-coated solid lipid nanoparticles (SLNs) encapsulating artichoke bract extracts, rich in CA and related phenolics, exhibited enhanced permeability, sustained release, and improved antioxidant and metal chelation capacities. In vivo, these SLNs significantly ameliorated cognitive deficits, reduced neuroinflammation and Aβ/tau pathology, and preserved hippocampal architecture in a sporadic AD mouse model [56]. Collectively, these studies underscore that nanoformulation technologies not only enhance the brain delivery of CA but also potentiate its multimodal neuroprotective mechanisms.

### 3.5. Targeted Delivery and Surface Modification

Latest innovations in drug delivery platforms have significantly enhanced the therapeutic efficacy of CA in AD by addressing its inherent challenges, such as limited bioavailability and low specificity to the brain. A noteworthy development involves the use of carbon quantum dots conjugated with CA and enveloped in microglial membranes (CDs-CA-MGs), specifically designed for intranasal delivery. This system has demonstrated inflammation-responsive release of CA, targeted brain accumulation in 5xFAD mice, and effective suppression of neuroinflammatory markers and Aβ-associated toxicity in both in vitro and in vivo settings, ultimately leading to improved cognitive performance [57]. Additionally, transferrin-functionalized liposomes encapsulating CA have been developed to facilitate receptor-targeted transcytosis across the BBB. These nanosystems, optimized through reverse-phase evaporation, ensure sustained release, high encapsulation efficiency, and effective inhibition of Aβ_1–42_ aggregation and fibril destabilization, as evidenced by Thioflavin T kinetics and TEM imaging. Collectively, these studies highlight the potential of precision-targeted nanoformulations in optimizing the delivery and therapeutic impact of CA in neurodegenerative diseases [58].

While each of these systems—nanoformulations, targeted delivery, and surface modifications—aims to enhance the bioavailability and therapeutic precision of CA, they possess distinct advantages and limitations. Nanoformulations enhance solubility and offer protection against degradation, yet they may lack targeting specificity. Targeted delivery systems, such as transferrin-mediated platforms, increase selectivity for the brain; however, they necessitate complex design and validation processes. Surface modifications, such as microglial coating, facilitate site-specific release and immune evasion but may unpredictably alter pharmacokinetics. The integration of these strategies may yield synergistic benefits by balancing efficacy with delivery precision.

### 3.6. Derivatives

The pharmacokinetic characteristics of CA and its derivatives have attracted significant scholarly interest, particularly concerning their permeability across the blood–brain barrier (BBB) and systemic bioavailability. Wang et al. (2022) [59] utilized an LC-MS/MS-based transwell assay with human brain microvascular endothelial cells to evaluate CA’s permeability. The study demonstrated that CA exhibited high passive diffusion (P_app = 2.15 × 10^−5^ cm/s) and a low efflux ratio (~1.05), indicating its effective ability to traverse the BBB. These findings substantiate CA’s potential as a candidate for central nervous system (CNS)-targeted therapies and validate its integration into delivery-focused strategies [59]. Further expanding the therapeutic profile of CA, Luo et al. (2021) [60] examined a modified derivative, CAPE-pNO_2_, within a diabetic AD model. This nitro-derivative displayed significant neuroprotective effects by activating Nrf2 signaling, enhancing Sirt1 and eNOS pathways, and reducing Aβ and p-tau accumulation. Its efficacy was confirmed both in vitro and in vivo, and it was notably reversed by selective Nrf2 inhibition, highlighting the compound’s redox-mediated multitarget action. These results position CAPE-pNO_2_ as a promising therapeutic scaffold for addressing AD in the context of metabolic comorbidities [60].

To provide a comprehensive summary of the experimental designs, targets, and outcomes associated with CA across different models of Alzheimer’s disease, all in vitro, in vivo, and delivery-based studies described in Section 3.1, Section 3.2, Section 3.3 are compiled in Table 1.

## 4. Discussion

CA has emerged as a promising neuroprotective agent in AD, with multiple studies supporting its ability to modulate key pathological features through diverse mechanisms. Based on in vitro, in vivo, and formulation research, CA exerts antioxidant, anti-inflammatory, anti-amyloidogenic, cholinergic, and mitochondrial protective effects, providing a strong rationale for further preclinical and clinical studies.

CA acts at the molecular level by modulating several critical neurodegenerative pathways. It enhances antioxidant defenses by activating Nrf2 and HO-1 and inhibits proinflammatory signaling cascades, including TNF-α, IL-6, NF-κB, and TLR4 [8,29]. These actions support neuronal survival and synaptic integrity via upregulation of neurotrophic markers, such as BDNF, SNAP-25, and PSD-95, and modulation of PI3K/Akt and MAPK signaling [42].

Restoring cholinergic neurotransmission is a central strategy for managing AD symptoms. CA and CA-rich extracts have demonstrated selective and dual inhibition of AChE and BChE, both of which are implicated in AD pathophysiology [19,20,21,22]. For example, Berdav propolis showed potent AChE inhibition, whereas extracts from *Laurus nobilis* and *Beta vulgaris* displayed combined effects on AChE and BChE [21].

CA exhibits significant radical scavenging capacity, suppresses oxidative stress, and modulates inflammatory mediators in neuronal models [27]. CA-enriched extracts from sorghum and coffee demonstrated protective effects against Aβ-induced ROS, lipid peroxidation, and neuroinflammation, even after gastrointestinal simulation [28,53].

In vitro and in vivo models have shown that CA reduces Aβ aggregation and tau phosphorylation and restores mitochondrial bioenergetics. For example, sorghum-derived extracts reduced pS199 and pT231 tau levels and improved mitochondrial membrane potential and ATP production in neuroblastoma cells [32]. Additionally, docking studies confirmed CA binding to Aβ42 fibrils, implicating anti-aggregation properties [55].

Multiple rodent and invertebrate AD models have validated CA’s neuroprotective potential. In scopolamine, AlCl_3_, and TMT-induced models, CA improved cognitive performance and reduced oxidative and inflammatory damage [44,45]. In genetic models such as ApoE−/− mice, *C. elegans*, and transgenic *Drosophila*, CA prolonged lifespan, enhanced memory, and reduced Aβ burden and apoptosis markers [47,50].

However, CA’s bioavailability after oral administration and BBB penetration remain major limitations to its wider use in AD therapy. Advanced nanocarriers, such as chitosan-MgO nanocomposites, SLNs, Tf-liposomes, and carbon dot conjugates, improve CNS delivery, stability, and anti-AD efficacy of CA [55,56,57,58]. For instance, CA-loaded liposomes not only inhibited Aβ aggregation but also disassembled preformed fibrils [59].

Despite substantial preclinical evidence supporting the neuroprotective role of CA, a significant limitation of numerous studies is their dependence on complex plant extracts, where CA is merely one of several active constituents. Although advanced analytical techniques confirm the presence and occasionally quantify CA, the observed biological effects cannot be definitively attributed to CA alone. Synergistic or antagonistic interactions with other polyphenols, alkaloids, or terpenoids within the extracts may confound mechanistic interpretations. Furthermore, only a minority of studies have employed pure CA in isolation, with even fewer conducting comparative analyses between isolated CA and whole-extract formulations. This complicates the assessment of dose–response relationships and obscures the specific contribution of CA to therapeutic outcomes. Future research should prioritize the use of standardized, purified CA preparations and include appropriate controls to disentangle its direct effects from those of coexisting phytochemicals. Additionally, well-designed pharmacokinetic and pharmacodynamic studies are necessary to delineate its bioavailability, metabolism, and potential synergy with other neuroprotective agents.

## 5. Conclusions

CA, a naturally occurring polyphenol prevalent in dietary and medicinal plants, demonstrates significant neuroprotective potential in the context of AD. This comprehensive review of in vitro, in vivo, and formulation-based studies highlights CA’s multitarget mechanisms, including its capacity to inhibit cholinesterases, scavenge ROS, modulate neuroinflammatory pathways, and interfere with amyloid and tau pathologies. Furthermore, evidence from experimental models underscores CA’s ability to preserve synaptic function and mitochondrial integrity, thereby supporting neuronal survival and cognitive function. Despite its pharmacological versatility, the clinical translation of CA is impeded by its limited oral bioavailability and BBB permeability. Recent advancements in nanotechnology, targeted delivery systems, and structural derivatives present promising strategies to overcome these limitations and enhance CA’s therapeutic efficacy. Future research should prioritize clinical validation, standardized formulations, and mechanistic elucidation in human-relevant models to facilitate the transition of CA from bench to the treatment of AD.

## Figures and Tables

**Table 1:** Summary of in vitro, in vivo, and formulation-based studies evaluating the neuroprotective effects of CA in Alzheimer’s disease.

Reference (No.)	Experimental Model/Method	Biological Target/Mechanism	Main Outcomes/Findings	Study Type
[19]	Methanolic extract of *Ononis natrix*	BChE	Potent BChE inhibition (IC_50_ = 9 µg/mL); CA confirmed active	In vitro
[20]	*Laurus nobilis* extract with moist heat processing	BChE	Significant inhibition (IC_50_ = 17.3 µg/mL); CA enriched by processing	In vitro
[21]	Propolis extract from Berdav region	AChE	IC_50_ = 3.4 μg/mL; CA showed selective AChE inhibition	In vitro
[22]	*Beta vulgaris* extract and docking	AChE/BChE	Moderate dual inhibition; CA confirmed via docking	In vitro
[23]	*Salvia fruticosa* extract and GSK-3β inhibition	GSK-3β	Inhibition through kinase pathway modulation; docking with CA	In vitro
[25]	*Sorghum bicolor* extracts on BE(2)-M17 cells	ROS, Aβ aggregation	↓ ROS, ↑ viability, anti-amyloid effect (up to 78%)	In vitro
[26]	*Blumea laciniata* extract (DPPH, ABTS)	Antioxidant enzymes	CA among compounds contributing to strong antioxidant activity	In vitro
[27]	*Camellia sinensis* (fermentation)	Antioxidant and AChE/BChE	Improved antioxidant and cholinesterase inhibition after fermentation	In vitro
[28]	*Rosmarinus officinalis* on HT22 cells	Aβ25–35, MMP9, MAPK14	Restored mitochondria, ↓ apoptosis, confirmed CA binding	In vitro
[29]	Indian propolis (PC-12 cells)	PTGS2, Oxidative markers	Dose-dependent antioxidant effects; CA derivative with COX-2 affinity	In vitro
[32]	*Sorghum* extracts on BE(2)-M17 cells	Tau, Mitochondria	↓ p-Tau, ↑ ATP; CA-rich extract reversed Aβ toxicity	In vitro
[33]	*Stachys* species screening	AChE, NMDAR	CA as dual ligand with stable dynamics; favorable ADMET	In silico
[34]	AChE-functionalized nanoparticles	AChE	CA as negative control; specificity validation	In vitro
[35]	Methanolic extract of *Sophora mollis*	AChE, Antioxidant	CA (45.2 ppm) showed IC_50_ = 75.96 μg/mL; neuroprotective via AChE inhibition and antioxidant activity	In vitro
[36]	*Huperzia serrata* formulation (NSP01) with HA, CA, ferulic acid	Glutamate, Aβ_1–42_, ERK1/2	CA synergized with HA, preserved neurons, activated ERK1/2; no additional AChE inhibition	In vitro
[37]	CA kinetic and docking study	AChE	Moderate inhibition (IC_50_ = 16.80 µM; Kᵢ = 12.42 µM); binds AChE active site residues	In vitro
[38]	CA–transferrin interaction (molecular dynamics)	Iron homeostasis, Oxidative stress	CA binds Htf iron pocket; suggests role in metal-regulated oxidative stress in AD	In silico
[39]	CA docking to ApoE4 (molecular simulations)	ApoE4	Strong binding (−101.9 kcal/mol) to ApoE4; potential for ApoE4-targeted AD therapy	In silico
[42]	TMT-induced rats + *Ixeris dentata*/CA	AChE, CREB, ChAT	↑ memory, ↑ CREB/ChAT expression; CA effective	In vivo
[43]	Scopolamine-induced mouse model + Divya-Medha-Vati	AChE, ROS, BDNF	↑ cognition, ↓ oxidative stress; CA among active compounds	In Vivo
[44]	Sodium azide rat model + *Mazus pumilus*	AChE, Aβ, Tau	↑ cognition, ↓ plaques/tangles; CA present via HPLC	In vivo
[45]	Scopolamine-induced mouse model + *Desmodium* extract	AChE, BChE, ROS	↑ cognition; docking supports CA action	In vivo
[46]	D-galactose + AlCl3 model + *Physalis extract*	Aβ, Tau, p38 MAPK	↓ amyloid/tau, ↓ inflammation; CA among components	In vivo
[47]	ApoE−/− mice + CA	Aβ, Inflammation, ABCA1	↓ Aβ, ↓ cytokines, ↑ memory; dual action	In vivo
[48]	Scopolamine-induced mouse model + *Acanthopanax* extract	AChE, Apoptosis	↑ cognition, ↓ AChE/apoptosis; CA interaction confirmed	In vivo
[49]	*Drosophila* APP/BACE1 model + Solanum extract	Aβ, AChE, MAO	↓ Aβ, ↑ behavior; CA-linked effects	In vivo
[50]	*C. elegans* Aβ model + CA	DAF-16/FOXO, ROS	↑ lifespan, ↓ Aβ, ↑ stress genes	In vivo
[51]	*Elaeocarpus* extract	TACE, AChE, APP	CA binds TACE; broad multitarget profile	In vivo
[52]	AlCl_3_-induced rats + peanut extract	AChE, TNF-α	↓ AChE, ↓ neuroinflammation; CA bioavailability enhanced	In vivo
[8]	Aβ_1–42_ mice + CA (50 mg/kg)	BDNF, Aβ, PI3K/Akt	↑ synaptic markers, ↓ inflammation	In vivo
[53]	Simulated digestion of coffee polyphenols	BChE	Green coffee → ↑ BChE inhibition; CA bioactive	In vivo
[54]	Metabolic syndrome rats + CA	TNF-α, IL-6, BDNF	↓ cytokines, ↑ BDNF; partial reversal	In vivo
[55]	STZ rat + CH/PLA/MgONCs	AChE, ATP, TNF-α	↑ memory, ↓ oxidative stress, CA binds Aβ42	Nanoformulation
[56]	STZ mouse + ART-SLNs	Aβ, Tau, TNF-α	↑ memory, ↓ Tau/Aβ; SLN ↑ brain delivery	Nanoformulation
[57]	5xFAD mice + CDs-CA-MGs	Aβ, IL-6, TNF-α	↓ Aβ, ↑ IL-10; CA release in inflammation	Targeted Delivery
[58]	Liposomal CA (Tf-conjugated)	Aβ aggregation	Sustained CA release, ↓ fibrils, ↑ BBB transport	Targeted Delivery
[59]	BBB model with CA transport assay	BBB permeability	↑ CA permeability, low efflux	Derivative
[60]	Diabetic mice + CAPE-pNO_2_	Aβ, p-Tau, Nrf2	↓ Aβ/Tau, ↑ Sirt1/Nrf2; multitarget protection	Derivative

## Data Availability

No new data were created or analyzed in this study. Data sharing is not applicable to this article.

## Supplementary Materials

The following supporting information can be downloaded at: https://www.mdpi.com/article/10.3390/medicina61081428/s1. Table S1: PRISMA 2020 Checklist [61].

## Abbreviations

The following abbreviations are used in this manuscript:

ABTS	2,2′-Azino-bis(3-ethylbenzothiazoline-6-sulfonic acid)
AChE	Acetylcholinesterase
AD	Alzheimer’s disease
APP	Amyloid precursor protein
BBB	Blood–brain barrier
BChE	Butyrylcholinesterase
BDNF	Brain-derived neurotrophic factor
CA	Caffeic acid
CAPE	Caffeic acid phenethyl ester
CAT	Catalase
ChAT	Choline acetyltransferase
COX	Cyclooxygenase
CREB	cAMP response element-binding protein
CRP	C-reactive protein
CUPRAC	Cupric ion reducing antioxidant capacity
DPPH	2,2-Diphenyl-1-picrylhydrazyl
eNOS	Endothelial nitric oxide synthase
GSH	Glutathione
IL	Interleukin
MAO	Monoamine oxidase
MAPK	Mitogen-activated protein kinase
MDA	Malondialdehyde
Nrf2	Nuclear factor erythroid 2–related factor 2
PI3K	Phosphoinositide 3-kinase
ROS	Reactive oxygen species
SIRT1	Sirtuin 1
SLNs	Solid lipid nanoparticles
SOD	Superoxide dismutase
STZ	Streptozotocin
Tf	Transferrin
TNF-α	Tumor necrosis factor alpha
